# Surveillance Training for Ebola Preparedness in Côte d’Ivoire, Guinea-Bissau, Senegal, and Mali

**DOI:** 10.3201/eid2313.170299

**Published:** 2017-12

**Authors:** Victor M. Cáceres, Sekou Sidibe, McKenzie Andre, Denise Traicoff, Stephanie Lambert, Melanie King, Ditu Kazambu, Augusto Lopez, Biagio Pedalino, Dionisio J. Herrera Guibert, Peter Wassawa, Placido Cardoso, Bernard Assi, Alioune Ly, Bouyagui Traore, Frederick J. Angulo, Linda Quick, STEP Working Group

**Affiliations:** Centers for Disease Control and Prevention, Atlanta, Georgia, USA (V.M. Cáceres, M. Andre, D. Traicoff, S. Lambert, M. King, A. Lopez, B. Pedalino, F.J. Angulo, L. Quick);; CDC Foundation, Atlanta (S. Sidibe);; African Field Epidemiology Network and Field Epidemiology Training Program, Dakar, Senegal (D. Kazambu);; Training Programs in Epidemiology and Public Health Interventions Network, Atlanta (D.J. Herrera Guibert);; Makerere University School of Public Health, Kampala, Uganda (P. Wassawa);; Instituto Nacional Saúde Pública, Bissau, Guinea-Bissau (P. Cardoso);; Institut National d'Hygiene Publique, Abidjan, Côte d’Ivoire (B. Assi); Centre des Opérations d’Urgence Sanitaire, Dakar (A. Ly); African Field Epidemiology Network, Field Epidemiology Training Program, Centre National d’Appui à la lutte contre la Maladie, Bamako, Mali (B. Traore)

**Keywords:** global health security, training, surveillance, STEP, SMS, Ebola, outbreak, zero-reporting, text message, training-in-service, field-based training, Côte d’Ivoire, Guinea-Bissau, Senegal, Mali, viruses

## Abstract

The 2014–2015 epidemic of Ebola virus disease in West Africa primarily affected Guinea, Liberia, and Sierra Leone. Several countries, including Mali, Nigeria, and Senegal, experienced Ebola importations. Realizing the importance of a trained field epidemiology workforce in neighboring countries to respond to Ebola importations, the Centers for Disease Control and Prevention Field Epidemiology Training Program unit implemented the Surveillance Training for Ebola Preparedness (STEP) initiative. STEP was a mentored, competency-based initiative to rapidly build up surveillance capacity along the borders of the at-risk neighboring countries Côte d’Ivoire, Mali, Senegal, and Guinea-Bissau. The target audience was district surveillance officers. STEP was delivered to 185 participants from 72 health units (districts or regions). Timeliness of reporting and the quality of surveillance analyses improved 3 months after training. STEP demonstrated that mentored, competency-based training, where learners attain competencies while delivering essential public health services, can be successfully implemented in an emergency response setting.

By January 2016, 2 years after the beginning of the epidemic of Ebola virus disease in West Africa, 28,616 cases and 11,310 deaths had been reported (*1*). Nearly all cases occurred in 3 countries (Guinea, Sierra Leone, and Liberia); however, several countries experienced Ebola importations, including Mali, Nigeria, and Senegal. Rapid response in Nigeria prevented catastrophic widespread Ebola transmission in one of the most densely populated areas in Africa (*2*). Containing and ultimately eliminating widespread transmission in the heavily affected countries required an unprecedented collaboration of global partners working closely with ministries of health (MOHs) in epidemiology and surveillance; this included laboratory support, infection prevention and control (including isolation), treatment, safe burials, risk communication, and training of local workers in each domain. A notable contribution to the response was the emergency implementation of the Surveillance Training for Ebola Preparedness (STEP) initiative, led by the Centers for Disease Control and Prevention (CDC) Field Epidemiology Training Program (FETP) unit, to rapidly build up surveillance capacity along border districts and regions in the 4 countries (Guinea-Bissau, Senegal, Mali, and Côte d’Ivoire) sharing land borders with the 3 heavily Ebola-affected countries. STEP was urgently needed because of the exponential human-to-human spread of Ebola, porous borders, massive seasonal population movements, and limited epidemiologic surveillance infrastructure.

CDC has a long history of assisting MOHs in building the capacity of their public health workforces. For over 35 years, CDC’s FETP has helped countries strengthen disease surveillance and epidemiology through mentored, competency-based training in which trainees attain competencies while delivering essential public health services (*3*). Integrated Disease Surveillance and Response (IDSR), a program CDC developed jointly with the World Health Organization (WHO) and widely adopted in Africa, provides guidelines and trainings to improve disease surveillance at local and district levels (*4*). STEP integrated the principles of FETP (mentored, training-in-service approach) with the IDSR framework to implement a 5-week, highly focused training with 2 goals: increase timeliness and quality of surveillance data reports, and increase the number of facilities reporting.

STEP received its funding from an Ebola-focused emergency US congressional appropriation. The time from funding availability to implementing partners to initiation of onsite training (including conceptual design, partnership formation, materials development and translation) was approximately 10 weeks. CDC led the implementing partnership consisting of MOHs in Guinea-Bissau, Côte d’Ivoire, Mali, and Senegal; the Training Programs in Epidemiology and Public Health Interventions Network (TEPHINET); the African Field Epidemiology Network (AFENET); and WHO (*5**,**6*). We report on the STEP experience in Guinea-Bissau, Senegal, Mali, and Côte d’Ivoire, highlighting successes, challenges, and lessons for the future. We also describe an initiative to implement daily, short message service (SMS) text-based reporting for suspected Ebola cases, an activity added to STEP training in response to an acute need for improved situational awareness along border districts (*7*).

## Methods

### Partner Collaboration

Each partner in the STEP initiative played a critical role. CDC led the overall initiative and provided technical expertise. TEPHINET, a global, professional network of FETPs, was responsible for recruiting and providing transport for 1–2 senior epidemiologists with field epidemiology expertise and language skills, who served as trainers and mentors in each country (e.g., Brazilian mentors in Guinea-Bissau, a Rwandan mentor in Côte d’Ivoire). AFENET, with its extensive experience with strengthening FETPs in Africa, was responsible for training logistics, including providing transport for participants, identifying and securing training venues, and translation and printing of materials. In each country, CDC partnered with the entity within the MOH responsible for disease surveillance: in Côte d’Ivoire, Institut National d’Hygiene Publique (INHP); in Guinea-Bissau, Instituto Nacional Saúde Pública (INASA); in Senegal, Centre des Opérations d’Urgence Sanitaire (COUS); and in Mali, Centre National d’Appui à la lutte contre la Maladie (CNAM). An MOH representative served as the point of contact, working closely with CDC to ensure country engagement, identify the appropriate training audience, and provide in-depth knowledge of the country’s surveillance system. Disease Prevention and Control Officers from the WHO Regional Office for Africa (AFRO) provided vital information about disease surveillance in-country.

### Country Engagement

Initial communication with country representatives about the training was conducted through CDC country offices where present (Côte d’Ivoire, Mali) and the High-Risk Unaffected Countries Team (a component of CDC’s Ebola response) (*8*). The project description document, curriculum plan, and country planning worksheet were shared as part of this initial communication, and customized in accordance with each country’s input. Countries sent letters inviting CDC and its partners to conduct the training, indicating the MOH’s point of contact, and including a list of proposed districts/regions and participants.

### Curriculum

The curriculum integrated classroom instruction with field assignments, mentorship, and SMS daily reporting to achieve STEP’s overarching goals. The project team finalized the proposed program objectives based on each country’s MOH planning discussion. The target audience was surveillance officers at the first level of the health system where data from local health facilities are aggregated and reported up. Materials from the IDSR and FETP library were adapted to the country context (e.g., surveillance infrastructure, notifiable disease list) and the urgent needs of the outbreak. The classroom component emphasized specific desired competencies to which each MOH had agreed during the initial country meetings. An Ebola case study, complementary field guidelines, and mentor guides were developed. All materials were created in English and translated into French and Portuguese.

### In-Country Training

STEP training was led by senior CDC epidemiologists with support from TEPHINET mentors and MOH and AFRO representatives. The training lasted 5 weeks and had 3 distinct components (Table 1). Two cohorts were trained in each country, except for Mali, where only 1 cohort was trained due to security constraints. 

**Table 1:** Surveillance Training for Ebola Preparedness 5-week program timeline

Week 1	Weeks 2–4	Week 5
Workshop 1	On-the-job fieldwork	Workshop 2
Interactive learning based on Integrated Disease Surveillance and Response	Data analysis and quality audit	Present results
Ebola virus disease, case investigation, and contact tracing	Surveillance summary report	Engage in continuing education on outbreak response, report writing, additional topics per local requirements
Surveillance system monitoring		Self-assess goal progress
Magpi (http://home.magpi.com) daily short message service reporting		Draft plan to improve local surveillance
Draft goals		

During Workshop 1, which lasted 5 days, participants engaged in interactive learning on IDSR, Ebola virus disease, investigation and contact tracing, surveillance system monitoring, and daily SMS zero-reporting. After Workshop 1, participants returned to their respective districts/regions for 3 weeks to review processes for surveillance data collection, data analysis, and disease notification. They completed 2 field projects: 1) conducting a data quality audit by visiting a minimum of 3 health posts in their district, and 2) drafting a surveillance summary report of nationally reportable diseases. During the 3 weeks of field assignments, participants were supported by TEPHINET mentors through site visits, phone calls, and emails. The final component of the training consisted of a 3-day workshop in the fifth week (Workshop 2), during which participants presented findings from the field to trainers and ministry officials, received feedback from the trainers, and developed plans for improving local surveillance.

### Daily SMS Zero-Reporting

Daily SMS zero-reporting was designed as a management tool to supplement, not replace, the MOHs’ existing systems for immediate reporting. The process allowed STEP participants to implement the principles of zero-reporting of suspected Ebola cases using Magpi, a cloud-based mobile data collection application that works with simple phones, smartphones, tablets, and computers (http://home.magpi.com). Zero-reporting means the reporting of the absence or presence of a disease or syndrome at a regular interval and is critical for the surveillance of a rapidly spreading infectious disease. The pilot implementation of daily SMS reporting in Guinea-Bissau has been previously reported (*7*). In Guinea-Bissau, Senegal, and Mali, 1 participant (the reporter) from each border district or region (and other districts/regions specifically requested by MOHs) was provided with a simple cell phone for sending daily SMS texts indicating the number of newly identified cases under investigation for Ebola in the previous 24 hours. System setup and SMS training occurred during Workshop 1. The countries generally used the standard WHO suspected Ebola case definition (Case Under Investigation): any person who has traveled to or stayed in a country that has reported >1 confirmed case of Ebola virus disease within <21 days of the onset of symptoms and who reports sudden onset of high fever and any of the following symptoms: headache, vomiting, diarrhea, anorexia/loss of appetite, lethargy, stomach pain, aching muscles or joints, difficulty swallowing, breathing difficulties, hiccups; or inexplicable bleeding/hemorrhaging; or who died suddenly and inexplicably.

The SMS text was received by a smartphone connected to the MOH office’s wireless network and uploaded automatically to the Magpi cloud in real time. An Epi Info cloud–Magpi bridge application (http://eicloudmagpibridge.codeplex.com) was used to extract collected data from the Magpi cloud. An Epi Info cloud data analytics application (http://www.cdc.gov/epiinfo/cloud.html) was used to generate tables, charts, and maps that were available on a real-time, web-based dashboard. CDC distributed to MOH points of contact a weekly summary indicating each district’s reporting rate for the preceding week and for the entire reporting period to date. Daily zero-reporting was closely monitored from the date of first report submission in each country through November 1, 2015, after which the risk of Ebola importation was very low due to the disease disappearing in affected countries.

### Evaluation Plan

The program evaluation plan consisted of 2 strategies: 1) a pretraining (baseline) and posttraining Surveillance Practices Self-Assessment (SPSA), and 2) a Predictive Evaluation framework (*9*) which linked STEP objectives to anticipated behavior changes on the job. At the beginning of Workshop 1, participants completed the baseline SPSA to provide data about their current work responsibilities, assessing whether participants met target audience criteria. The respondents were also asked about the content and quality of surveillance reports (e.g., “What percentage of routine summary surveillance reports include tables, graphs, or maps?”). After 3–6 months, an evaluator would conduct in-person interviews to reassess their surveillance practices and elicit information about key competencies attained from the training course, deliverables achieved posttraining, progress made toward their goals, and other changes resulting from STEP.

The Predictive Evaluation framework approach uses specific performance objectives defined by the country and establishes a committee to design workshop content. Stakeholders predict the new or changed behaviors they expect to see after a successful workshop. At the end of the workshop, participants develop statements describing specific actions they intend to do with their new knowledge, and their statements are compared with the stakeholders’ expectations. In accordance with the Predictive Evaluation framework, participants were instructed at the end of Workshop 1 to draft 1–2 goals describing specific actions they would take upon returning to the workplace, and to align these goals with classroom learning objectives. The STEP staff analyzed goal statements for quality, based on the four criteria of specific, observable, impactful, and directly related to the training content. Participants were asked about their progress toward their goals upon return from their fieldwork for Workshop 2.

## Results

The implementation of STEP in Côte d’Ivoire, Guinea-Bissau, Senegal, and Mali occurred during an 8-month period, beginning with the first training in Côte d’Ivoire on January 12, 2015, and ending with the last training in Mali on August 19, 2015. STEP trained 185 participants from 72 health units (61 districts and 11 regions) in these 4 countries (Table 2). Among the participants were 47 district surveillance officers, 45 district medical officers, and other district-level staff who were responsible for frontline analysis and reporting of surveillance data. Although STEP was primarily designed for surveillance officers at the first level of the health system, 42 regional surveillance officers also participated in the training to reinforce the work of the district-level surveillance officers whom they supervised.

**Table 2:** Surveillance Training for Ebola Preparedness training information for 4 countries in West Africa, 2015*

Country	Training dates	No. cohorts	No. participants	No. health units, districts or regions
Côte d’Ivoire	Jan 12–Mar 18	2	54	25 districts
Guinea-Bissau	Jan 19–Mar 25	2	53	11 regions
Senegal	Apr 7–Jun 10	2	52	21 districts
Mali	Jul 20–Aug 19	1	26	15 districts
Total		7	185	61 districts, 11 regions

*The number of Ebola-related deaths in West Africa peaked during October–December 2014. †For purposes of this program, the 6 communes in Bamako, Mali, are counted as 6 distinct health districts yielding a total of 15.

The results of the baseline SPSA confirmed that the appropriate participants had been recruited, with 155 (84%) of the 184 participants responding that they performed surveillance activities as part of their routine work when they began the training. Participants’ responses to questions about current reporting practices indicated that pretraining surveillance practices were not optimal (Table 3).

**Table 3:** Overall baseline Surveillance Practices Self-Assessment results from Surveillance Training for Ebola Preparedness program for 4 countries in West Africa, 2015*

	No. (%) participants			
Surveillance practice	Côte d’Ivoire n = 54†	Guinea-Bissau n = 52†	Senegal n = 52	Mali n = 26
Participant performs surveillance work as part of routine work responsibilities	53 (98)	39 (75)	43 (83)	20 (77)
Most routine surveillance reports submitted to the district/region:‡				
Were submitted on time	43 (80)	25 (48)	34 (65)	19 (73)
Were complete	34 (63)	17 (33)	36 (69)	20 (77)
Contained data on EVD indicating its presence or absence	37 (69)	11 (21)	19 (37)	20 (77)
Most summary surveillance reports developed by the participants:				
Included tables, graphs, or maps	14 (26)	5 (10)	6 (12)	12 (46)
Were analyzed using computer software	19 (35)	11 (21)	12 (23)	15 (58)
Included interpretations of the data	16 (30)	9 (17)	13 (25)	16 (62)
Included analyzed case-based data	5 (9)	4 (8)	11 (21)	15 (58)

*EVD, Ebola virus disease. †One participant did not complete assessment.  ‡Most indicates >50%.

**Figure 1:**
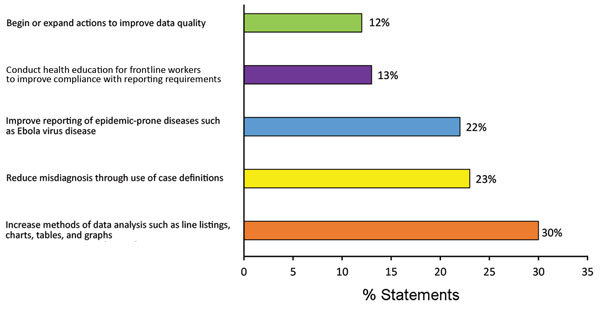
Distribution of 307 goal statements drafted by participants in Surveillance Training for Ebola Preparedness program in 4 countries in West Africa, categorized by related objective, January–August 2014.

The 307 goal statements that the 185 participants drafted for the Predictive Evaluation were categorized by the learning objective with which they are most closely associated (Figure 1). Examples of goal statements included the following:

• “After the data [are] collected, I will ensure that I do an analysis with diagrams, tables, and graphs, and present it to staff, service supervisors (major) to show the utility of the data and to altogether improve the data.”

• “I will encourage providers to make and transmit the report on time, and make a telephone reminder.”

• “[I will] reduce wrong diagnosis through the use of definition of cases by training health specialists in this field.”

Across countries, participants consistently demonstrated strong intent to improve the methods used for data analysis. Reducing misdiagnosis through the use of standard case definitions and improving reporting of epidemic-prone diseases were also frequently declared goals. Participants were less likely to make plans to improve reporting compliance or data quality. During the follow-up assessment during Workshop 2, most (110/133, 83%) sampled participants reported achieving (61 participants) or making significant progress toward (49 participants) their goal during 3 weeks of fieldwork (Table 4).

**Table 4:** Participant-reported goal progress during fieldwork for Surveillance Training for Ebola Preparedness program in 4 countries in West Africa, 2015

Progress toward >1 goal	No. (%) participants				
	Côte d’Ivoire, n = 54	Guinea-Bissau, n = 26	Senegal, n = 26	Mali, n = 26	Overall, n = 133
Achieved goal	28 (52)	10 (38)	10 (38)	13 (50)	61 (46)
Significant progress toward goal	18 (35)	9 (35)	12 (46)	10 (38)	49 (37)
Some progress toward goal	2 (4)	5 (19)	3 (12)	3 (12)	13 (10)
No progress toward goal	0	2 (8)	0	0	2 (2)
Forgot/lost goal	0	0	0	0	0
No response	6 (11)	0	1 (4)	0	8 (6)

Due to resource constraints, the team was only able to conduct the posttraining SPSA in Côte d’Ivoire (Table 5). Three months after the training was completed, the SPSA was readministered to 21 respondents from Côte d’Ivoire, with 1–2 graduates from each of the districts bordering Ebola-affected countries assessed. Surveillance practices improved in several ways between the onset of STEP and the 3-month posttraining follow-up. A substantial number of participants reported taking actions to strengthen the data flow from health facilities. All 21 participants (100%) reported working with health facility staff to strengthen awareness of case definitions, with 18 (86%) participants stating they had provided flyers with case definitions and distributed disease notification sheets to all health centers. We also found very little tolerance for late reporting, with 20 (95%) respondents stating they routinely follow up via phone call, SMS, or a personal visit with health facilities that do not report on time. Eighteen (86%) participants submitted weekly surveillance reports. Ten respondents (48%) reported training others in data analysis techniques or using analysis methods themselves to improve surveillance.

**Table 5:** Surveillance Practices Self-Assessment results before and 3 months after Surveillance Training for Ebola Preparedness program, Côte d’Ivoire Border District*

Surveillance practice	No. (%) participants	
	Before program, n = 21†	3 mo after program, n = 21
Participant performs surveillance work as part of routine work responsibilities	20 (95)	21 (100)
Most routine surveillance reports submitted to the district/region:‡		
Were submitted on time	19 (90)	20 (95)
Were complete	16 (76)	18 (86)
Contained data on EVD indicating its presence or absence	17 (81)	10 (48)
Most summary surveillance reports developed by the participants:		
Included tables, graphs, or maps	5 (24)	10 (48)
Were analyzed using computer software	7 (33)	14 (67)
Included interpretations of the data	5 (24)	13 (62)
Included analyzed case-based data	2 (10)	3 (14)

*EVD, Ebola virus disease. †The 21 respondents are a subset of the initial 54 participants. ‡Most indicates >50%.

**Figure 2:**
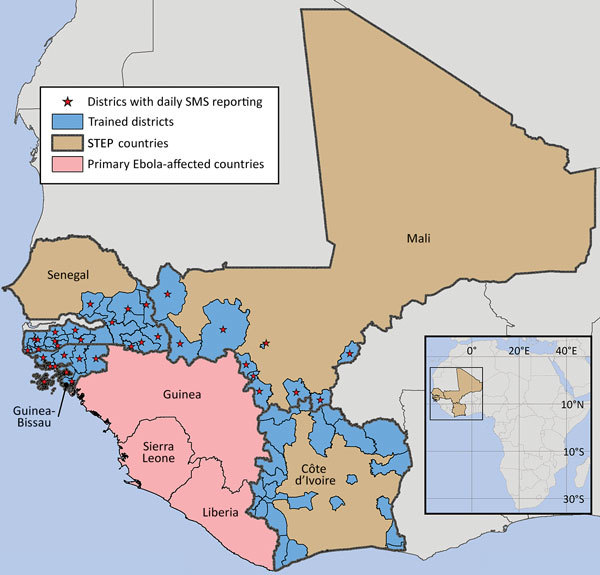
Districts and regions in 4 countries in West Africa participating in program training and daily SMS zero-reporting, 2015. The city of Bamako in Mali is administratively divided into 6 discrete communes, each equivalent to 1 health district. These are too small to individually illustrate on the map, so only Bamako, comprising all 6 communes, is shown. STEP, Surveillance Training for Ebola Preparedness; SMS, short message service. Map created by Andrew Berens. Sources: Global Administrative Areas (http://gadm.org); ERSI Data & Maps 2005.

**Figure 3:**
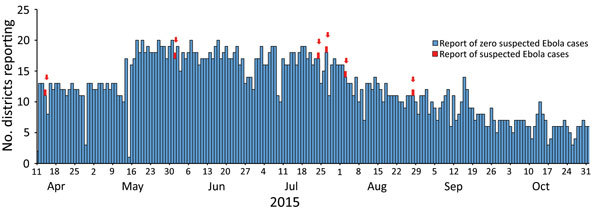
Number of districts reporting suspected cases of Ebola per day, Senegal, April 11–November 1, 2015 (n = 20).

Daily zero-reporting for suspected Ebola cases was implemented in 3 of the high-risk border countries; this included 13 sites in 11 regions and 1 national laboratory in Guinea-Bissau, 20 sites in 20 districts in Senegal, and 25 sites in 15 districts in Mali (Figure 2). The setup of the phones and SMS reporting system during Workshop 1 of the training took <8 hours in each country. Mean reporting rates among the countries ranged 53%–68% (Table 6), with slightly lower rates for countries reporting over a longer time (suggesting that reporting drops off with time). Eight suspected Ebola cases, 6 in Senegal (Figure 3) and 2 in Mali (not shown), were detected through the SMS system during the reporting periods, but none was confirmed.

**Table 6:** Daily zero-reporting rates for suspected Ebola cases using short message service texting for Surveillance Training for Ebola Preparedness program in 4 countries in West Africa, 2015

Country	No. reporters	Reporting dates	No. days	Mean reporting rate (range), %
Guinea-Bissau	14	Jan 24–Nov 1	282	53 (22–78)
Senegal	20	April 1–Nov 1	215	65 (23–93)
Mali	15	July 25–Nov 1	100	68 (24–98)

## Discussion

The STEP initiative successfully completed its emergency mission to scale up border preparedness to mitigate potential spread of disease from Ebola virus–affected countries. The training was highly valued and well accepted by the MOHs that received it. The evaluation suggested important changes in the self-reported work behaviors of several participants. At 83%, the percentage of participants reporting either substantial progress or goal completion in our study was higher than that reported in an earlier study (*10*). The rate of daily SMS-text zero-reporting, albeit declining with time, demonstrated the feasibility of this technology for active monitoring of suspected Ebola cases several months post-STEP training.

Little has been published on real-time training during disasters, emergencies, and disease outbreaks for healthcare or public health professionals. Historically, workforce trainings for disaster, emergency, and outbreak response efforts have targeted clinical health professionals to identify, diagnose, and treat affected persons in healthcare settings. Published trainings primarily used exercises, simulations, and online certification of healthcare workers for purposes of preparedness and response planning either hypothetically or in anticipation of a real event. The unprecedented magnitude and severity of the 2014–2015 Ebola epidemic in West Africa required a novel training approach focused on the public health workforce’s emergency response during the epidemic. It is important to understand the lessons learned and limitations of our approach. These lessons can be divided into the areas of MOH political buy-in, preparatory country visits and planning, multilevel partnerships, and training and curriculum approach.

Political buy-in was a challenge easily met in the emergency context of the Ebola virus epidemic. Two of the countries (Mali and Senegal) had previously experienced Ebola virus importations, and there was general recognition of the need to fortify porous country borders that were susceptible to Ebola virus spread. It was vital that the formal letter of invitation from each MOH recognize the multilateral partnership involved in the technical assistance as well as identify a principal MOH point of contact. The contact, generally a high-level decision maker, was key to leading in-country efforts such as identifying dates and venues for training, prioritizing districts and participants, and coordinating logistics planning with partner organizations. Planning was greatly facilitated in countries in which CDC had an office. In countries with no office, CDC’s Ebola Response High-Risk Unaffected Countries Team provided valuable support through its in-country deployed field staff. A 2-day preparatory in-person visit by CDC FETP and partner staff with each MOH was important to ensure a common understanding of training objectives, the appropriate STEP participants, and the surveillance context in each country.

The collaboration of CDC FETP with many of its longstanding partners (TEPHINET, AFENET, WHO [in-country and AFRO]) was key to the speedy recruitment of mentors and handling of logistics; the project team members had previously worked together and understood administrative mechanisms for moving financial resources, participants, and mentors. Also, these organizations could more readily ensure that decisions were consistent with the FETP approach of field-based, mentored training.

We tailored the training curriculum to the requirements of the emergency within each country’s context. We supplemented STEP classroom instruction with a continuum of activities (including group work, goal statements, action plans, field assignments, mentor supervision) that have been shown in previous evaluation research to be associated with posttraining work application (*10*). Participants enhanced existing skills and developed new ones to identify problems affecting disease surveillance systems in their districts and to propose practical solutions. The STEP approach directly linked technical expertise about surveillance and Ebola to country priorities and performance-based learning. We believe that this approach, supported by quality mentorship, was a key factor of success and is applicable to other diseases and surveillance efforts.

This program had several important limitations and challenges. We had insufficient resources to conduct posttraining evaluation in all 4 countries. Although the evaluation in Côte d’Ivoire was encouraging, the interpretation is limited because the data were self-reported and nonrandomized, and we do not know how long the positive work behaviors continued. We had also hoped to implement SMS text–based reporting in all 4 countries. In the 3 countries that implemented the system, we were encouraged by the generally high rates of reporting (Table 6). The system took only a few hours to set up in each country and worked without major disruptions. Working in countries with different official languages also presented challenges, both in timely translation of materials and in the recruitment of mentors with appropriate language skills. Travel to hard-to-reach areas and situational awareness of security-related developments were mitigated by having MOH supervisory staff accompany mentors on site visits and by communicating closely with the embassies.

The Ebola crisis brought to light the large gap in the number of epidemiologists needed in West Africa. In addition, we noted the lack of epidemiologic skills at the district (operational) level. In most countries, these staff are responsible for aggregating and reporting surveillance data and often are the first with the opportunity to analyze, communicate, and respond to local events. The Ebola epidemic underscored the importance of WHO’s International Health Regulations (IHR 2005) and the Global Health Security Agenda (GHSA) (*11*). The GHSA, started in 2014, is an “international collaboration that aims to support all countries in meeting IHR regulations and ensuring global health security” (*12*). One of the major activities of GHSA is to support workforce development activities to better prevent, detect, and respond to public health emergencies (*13*). With the conclusion of STEP and the Ebola epidemic, CDC’s FETP unit is building on the work we report here by continuing to work with MOHs throughout the region to build sustainable epidemiologic and surveillance capacity through implementation of the FETPs-Frontline program. FETPs-Frontline targets district-level surveillance officers for a 3-month competency-based training; it has been conducted in 14 countries in West Africa and recently expanded to other parts of the world. The experience of STEP demonstrates that rapid scale-up of surveillance capacity and daily zero-reporting in the midst of an epidemic can be successfully executed by leveraging established partnerships, simple technologies, and mentored, field-based training.
