# Correction: Vol. 23, Supplement

**DOI:** 10.3201/eid2401.C22401

**Published:** 2018-01

**Keywords:** errata, erratum, correction

The name of author Melanie E. King was incorrectly listed and several items in the text were unclear in Surveillance Training for Ebola Preparedness in Côte d’Ivoire, Guinea-Bissau, Senegal, and Mali (V.M. Cáceres et al.). The article has been corrected online (https://wwwnc.cdc.gov/eid/article/23/13/17-0299_article).

